# Ethics and Antimalarial Drug Resistance

**DOI:** 10.1007/978-3-030-27874-8_4

**Published:** 2020-04-06

**Authors:** Phaik Yeong Cheah, Michael Parker, Nicholas P. J. Day

**Affiliations:** 2 Monash Bioethics Centre, Monash University, Melbourne, VIC Australia; 3 Monash Bioethics Centre, Monash University, Melbourne, VIC Australia; 4 Mahidol Oxford Tropical Medicine Research Unit (MORU), Faculty of Tropical Medicine, Mahidol University, Bangkok, Thailand; 5 Centre for Tropical Medicine and Global Health, Nuffield Department of Clinical Medicine, University of Oxford, Oxford, UK; 6 The Ethox Centre, Nuffield Department of Population Health, University of Oxford, Oxford, UK; 7 Wellcome Centre for Ethics and Humanities, Nuffield Department of Population Health, University of Oxford, Oxford, UK

## Abstract

There has been impressive progress in malaria control and treatment over the past two decades. One of the most important factors in the decline of malaria-related mortality has been the development and deployment of highly effective treatment in the form of artemisinin-based combination therapies (ACTs). However, recent reports suggest that these gains stand the risk of being reversed due to the emergence of ACT resistance in the Greater Mekong Subregion and the threat of this resistance spreading to Africa, where the majority of the world’s malaria cases occur, with catastrophic consequences. This chapter provides an overview of strategies proposed by malaria experts to tackle artemisinin-resistant malaria, and some of the most important practical ethical issues presented by each of these interventions. The proposed strategies include mass antimalarial drug administrations in selected populations, and mandatory screening of possibly infected individuals prior to entering an area free of artemisinin-resistant malaria. We discuss ethical issues such as tensions between the wishes of individuals versus the broader goal of malaria elimination, and the risks of harm to interventional populations, and conclude by proposing a set of recommendations.

## The Problem, Context and Background

Malaria is the most important parasitic disease of man. It remains a major cause of death in tropical countries, and an important cause of illness , particularly in childhood . There were an estimated 435,000 deaths from malaria in 2017, of which over 90% were in Africa (Global Malaria Programme, World Health Organization 2018). Although there has been impressive progress in malaria control and treatment over the past two decades in recent years progress has stalled and there has been a resurgence of malaria in Southeast Asia, where antimalarial drug resistance is increasingly prevalent (Global Malaria Programme, World Health Organization 2018). One of the most important factors in the decline of malaria related mortality since the 1990s has been the development and deployment of highly effective treatment in the form of artemisinin-based combination therapies (ACTs) (Bhatt et al. 2015). ACTs are currently the mainstay of antimalarial treatment throughout the world, recommended by the World Health Organization as the first line treatment globally for falciparum malaria (World Health Organization 2016a). Their widespread deployment, along with the expanded use of insecticide treated bed nets, accounts for a large part of the reduction in malaria deaths in Africa over the past decade (White et al. 2014).

The effectiveness of current and future interventions are, however, at risk from the emergence of new forms of drug resistance. In the early 2000s malaria parasites that were partially resistant to the artemisinins emerged in Western Cambodia. The problem was identified and characterised in 2008 (Dondorp et al. 2009; Noedl et al. 2008). The hallmark of infection by these resistant parasites was slow parasite clearance rather than outright treatment failure (Dondorp et al. 2009). These slow clearing infections were associated with mutations in the *PfKelch13* gene, with multiple *PfKelch13* mutations described (though each parasite only carried one) (Ariey et al. 2014). By 2014 slow clearing malaria infections caused by *PfKelch13* mutation-carrying artemisinin resistant parasites could be found in Myanmar, Laos, Thailand, Vietnam and Yunnan province in China, and by 2016 *Pfkelch13* mutants were identified in Arunachal Pradesh state in northeastern India (Ashley et al. 2014; Tun et al. 2016; Mishra et al. 2016).

The initial response to the emergence of artemisinin resistance had two aspects. The major concerns were that: i. artemisinin resistance might lead to or combine with partner drug resistance resulting in resistance to ACTs. That is, to the loss of the drug combinations that are the mainstay of malaria treatment; and ii. that were this to be the case, artemisinin resistance and then ACT resistance then has the potential to spread from Southeast Asia to Africa, where the majority of the world’s malaria cases occur, with catastrophic consequences. There are precedents for this latter concern; in the last 60 years first chloroquine resistance and then sulfadoxine-pyrimethamine resistance arose in Western Cambodia and subsequently spread to Africa, leading to millions of deaths (Verdrager 1986; Roper et al. 2004; Trape et al. 1998).

Recognizing the risks and consequences of spread to Africa, the World Health Organization initially developed a plan to contain rather than eliminate the problem (World Health Organization 2011). However, this approach was criticized by some at the time who called for initiatives aimed at eliminating all malaria in the Greater Mekong Subregion, on the grounds that the resistant parasites were in fact already at that stage contained (Dondorp et al. 2017). They argued that the view that elimination would be an appropriate strategy was supported by mathematical modelling that showed that as malaria was controlled and transmission fell, the proportion of infections that were resistant would increase – the ‘last man standing’ would be resistant (Maude et al. 2012). This modelling suggested that resistance could not be eliminated without eliminating all malaria in the affected regions.

Bolstered by advocacy for global malaria eradication by the Bill and Melinda Gates Foundation, the WHO did eventually change its policy from containment to one of elimination (Global Malaria Programme WHO 2015), but the discovery through molecular studies that *PfKelch13* mutations had arisen spontaneously and independently multiple times within the Greater Mekong Subregion (GMS) led to what some saw as a reduction in the urgency to eliminate malaria to prevent the ‘spread’ of resistant parasites (Takala-Harrison et al. 2015). Surveillance for resistance and an aim to eliminate malaria *everywhere* was now the policy of WHO (World Health Organization 2017; Global Malaria Programme WHO 2016). This necessarily spread resources more widely and reduced the focus on drug resistance. Despite this there has been considerable investment in malaria control and elimination efforts in the GMS, with at least initially substantial reductions in malaria transmission (Dondorp et al. 2017). These successes have, however, to some extent masked the continued threat of increasingly drug resistant malaria parasites emerging and spreading in the region – as the mathematical modelling had predicted (Maude et al. 2009) – with outbreaks of artemisinin-resistant malaria occurring in areas previously considered malaria free (Imwong et al. 2015).

In the 10 years since the first description of artemisinin resistance the concern that ACT resistance and failure would develop has come to pass, with rising mefloquine resistance on the Thai-Myanmar border and piperaquine resistance in Cambodia, Thailand, Southern Laos and Viet Nam (Phyo et al. 2016a; Amaratunga et al. 2016; Imwong et al. 2017). Recent evidence that most of the resistance to dihydroartemisinin-piperaquine is due to the geographical *spread* of a particularly fit artemisinin-resistant parasite clone which has picked up piperaquine resistance has rekindled the debate about whether urgent focus should be placed on an accelerated effort to eliminate artemisinin-resistant malaria in Southeast Asia, with the aim of preventing the spread of ACT resistance to Africa (World Health Organization 2016b).

Although the global pipeline for new malaria drugs in development is healthier than it has been for decades, all the most promising candidates (schizonticidals, that kill the asexual blood stage of the parasite which causes the clinical manifestations of malaria) are at least five years away from being available in the market (Phyo et al. 2016b). The RTS,S/AS01 malaria vaccine which recently received a favourable scientific opinion from the European Medicines Agency (EMA) is only partially protective (Vandoolaeghe and Schuerman 2018). This means that protecting the efficacy of the currently available antimalarial drugs is of global importance. The spread of ACT resistance to Africa would threaten the loss of millions of lives, especially those of young African children. This will be a global health issue – untreatable malaria worldwide arising from resistance in Southeast Asia. A problem arising in a specific location that has the potential to threaten global health is not unique to malaria. Many ‘global health problems’ arise in low-income settings, for example outbreaks of Ebola in West Africa and Zika in South America, and are recognized as having worldwide implications. In the case of Ebola and Zika the World Health Organization formally declared a ‘ Public Health Emergency of International Concern’ (PHEIC), but for the emergence of artemisinin resistant malaria in Southeast Asia WHO has as of 2018 declined to do this despite some experts calling for it to do so (World Health Organization 2016b; Talisuna et al. 2012).

### How Should the Problem of Artemisinin Resistant Malaria be Tackled?

There is now broad agreement amongst experts that to prevent the spread of artemisinin-resistant *P. falciparum* it is necessary to completely interrupt *P. falciparum* transmission (Maude et al. 2009), and that a programme of accelerated malaria elimination is warranted in the GMS and surrounding areas. The scientific consensus is that a combination of strategies is required to achieve this (Dondorp et al. 2017; World Health Organization 2017). These include:

1. *Ongoing* *surveillance* with a network of village malaria workers (VMWs) in endemic areas trained and equipped to provide early detection and treatment of malaria cases.
2. *Targeting of the asymptomatic malaria reservoir* in so called malaria ‘hotspots’, identified through surveys of healthy individuals employing highly sensitive methods of parasite detection such as large volume quantitative PCR and highly sensitive rapid diagnostic tests (RDTs). This may take two forms:
  - *Mass drug administration* (MDA) -WHO agrees that targeted mass antimalarial drug administration may play an important role in malaria elimination (World Health Organization, Global Malaria Programme 2015a).
  - *Mass screening and treatment* (MSAT) using novel highly sensitive diagnostics.
3. *Vector control* with insecticide treated bed nets, despite these being less effective in the GMS than in Africa because of the biting habits of many of the vector species (biting in the forest rather than in houses, and in the early evening or morning).
4. *Targeting ‘source’ populations* such as forest workers and migrants, rather than ‘sink’ populations secondarily affected. This requires an understanding of transmission dynamics and population movements – important in the GMS where cross border movement/migration is common.
5. *Mandatory screening* may be necessary of possibly infected individuals entering an area free of artemisinin-resistant malaria (Houston and Houston 2015; Tatarsky et al. 2011).
6. Use of *effective antimalarial treatments*. Most currently approved ACTs consist of only two drugs (a fast acting short half-life artemisinin and a longer half-life partner drug) and are vulnerable to the development of resistance. The testing and deployment of new triple artemisinin-based combination therapies (TACTs) has been recommended, and several of these are currently being tested in the GMS and beyond (Dondorp et al. 2017).

In the face of the global threat posed by increasing ACT resistance, there is therefore now an emerging expert consensus that the combination of strategies outlined above is the most effective way of halting or slowing its international spread provide strong ethical arguments for their rapid adoption. Each of the interventions listed above present a wide range of interconnected challenges – including, scientific, technological, governmental, economic and ethical – all of which will need to be overcome if the elimination of malaria in the region is to be achieved. In addition to these practical scientific, and political challenges, the success of each of the interventions also depends upon the addressing of a number of important practical ethical questions, which need to be taken into account in their design and implementation. In the next section, we outline some of the most important practical ethical issues presented by each of the interventions proposed above.

## Practical Ethical Issues Arising in These Interventions

### Ongoing Surveillance

In many countries in the Greater Mekong Subregion, networks of VMWs are the cornerstone of malaria surveillance and the delivery of malaria-related interventions. These networks are usually run either directly by the national malaria control programmes (NMCPs) or by NGOs working with the NMCPs, but may also be put in place by private providers such as companies running palm oil plantations. The coverage of such networks has increased impressively in many areas in SEA, particularly in border areas, conflict zones and areas underserved by government health programmes. VMWs are consulted by villagers suffering from fever, and are equipped with RDTs and antimalarial treatments. Where a substantial proportion of febrile illnesses are indeed caused by malaria VMWs are a valuable resource for the local population. However, the success of malaria control and elimination efforts increasingly means that a diminishing proportion of the febrile illnesses they encounter are caused by malaria. Unfortunately, VMWs are not usually equipped to deal with these alternative causes of illness. This means that as malaria rates decline there is a risk that villagers will cease consulting VMWs as more of them are told that because their fever is not clinical malaria infection, no diagnosis or treatment of the cause of their illness is available or offered. Unless these village workers are retrained for a wider role as ‘village *health* workers’ able to manage other febrile illnesses or simple primary health problems, they will become increasingly irrelevant and demotivated. The consequences of this would be diminishing effectiveness of the malaria surveillance network itself, at the point in the elimination process when it is most needed and, in the absence of a wider role, for the goal of malaria elimination becoming a disincentive for the VMWs (and NMCPs), for many of whom being a VMW is a source of their livelihood. This suggests that, even if the elimination of malaria is the primary goal, there are strong arguments in favour of the provision of resources for access to health care beyond malaria in the region.

### Mass Drug Administration (MDA)

MDA in the context of malaria elimination consists of mass treatment with a schizonticide to kill the asexual blood stage of the parasite which causes the clinical manifestations of malaria combined with a transmission blocking drug to kill gametocytes. Giving such treatment to all members of a community should eliminate the asymptomatic parasite reservoir and speed up the interruption of malaria transmission. Where and when it is warranted has been the subject of much debate, but the consensus is that MDA should be targeted at communities with high transmission and a large asymptomatic reservoir (World Health Organization 2011; von Seidlein and Dondorp 2015). This requires a functioning surveillance system to identify such communities, with surveys of healthy individuals with highly sensitive tests to estimate accurately the scale of the asymptomatic reservoir.

There are a number of important ethical considerations when determining when and where to use MDA. The first of these arises out of the fact that MDA by its nature involves administering drugs to individuals who will not benefit *directly* from the treatment, i.e. to healthy people in the interests of the wider community and the broader goal of elimination. In the case of transmission blocking drugs this is the entire community, and for the schizonticidal drugs this is the substantial proportion of the community who are not infected with malaria parasites. However, if the MDA is effective and malaria is eliminated from the area, all individuals will benefit *indirectly* by living in a malaria free community. In the GMS the schizonticide currently used in MDA is dihydroartemisinin-piperaquine (DP), which in the treatment of malaria is considered a safe drug. Studies of the safety of DP in this context have shown that DP is safe (Tripura et al. 2018), but widespread deployment of DP exposes much larger numbers of individuals such that its rare but serious side effects may occur (Cheah and White 2016). The transmission blocking drug currently used in MDA is primaquine, which targets the transmissible sexual gametocytes not killed by the schizonticide but has little or no impact on the asexual parasites which cause disease. Primaquine is an oxidative drug which causes haemolysis (the rupture of red blood cells) in G6PD-deficient individuals (median 8% of the population in malaria endemic areas) when given in the large doses needed to radically cure vivax malaria (killing the hypnozoites in the liver) (Howes et al. 2012). However to kill gametocytes (rather than cure malaria) a single much lower dose is required, one considered safe to be administered to all individuals without prior G6PD testing (World Health Organization, Global Malaria Programme 2015b; Bancone et al. 2016).

A second ethically significant consideration is a worry that there is a risk that with the widespread deployment of antimalarial drugs in MDA the resulting increased drug pressure may itself lead to drug resistance, particularly in the case where elimination is not achieved. It has been argued on theoretical grounds that this is unlikely, but the risk however low highlights the importance of achieving elimination in areas where MDA is deployed (White 2017). This suggests that the initiation of MDA is only ethically justified where there is a genuine commitment to complete the elimination task. Once the process of MDA has begun, important ethical issues are presented relating to the question of when such an initiative should be ended.

Thirdly, the effectiveness of MDA is predicated upon high population coverage (World Health Organization 2017; Newby et al. 2015). Achieving this is a challenge for several reasons: explaining the rationale for taking antimalarials when asymptomatic can be difficult in the absence of an understanding of the underlying scientific concepts, target communities are often remote with poor access and populations can be highly mobile. For these reasons, effective community engagement efforts are essential, so that individuals are informed of the risks and benefits of malaria elimination efforts in general and MDA in particular (Adhikari et al. 2016; Peto et al. 2018). For effective community engagement those implementing MDA need to understand and adapt the information they provide and the form of the engagement they adopt, to the cultural and practical requirements of each community. Engagement with community leaders is essential, and coverage can be promoted by offering healthcare alongside MDA (Sahan et al. 2017; Pell et al. 2017). Effective community engagement may also minimize risks of coercion or counterproductive misunderstanding of the aims of the public health authorities (Parker and Allen 2013). The ethical issues around these concerns are similar in some respects to those encountered in the context of vaccination campaigns.

### Mass Screening and Treatment (MSAT)

Mass screening and treatment of village populations has been suggested as an alternative strategy to MDA for speeding up malaria elimination. Its advantage over MDA is that only those individuals with proven asymptomatic malaria infection will be exposed to antimalarial drugs and their attendant risks, negating many of the ethical concerns described in the MDA section above. However, the likely success of this is limited by the sensitivity of the tests available for detecting low levels of parasites in the blood. Because the current tests are laboratory based there is inevitably considerable delay between sampling and result, which appears to limit the effectiveness of MSAT (von Seidlein 2014). Highly sensitive rapid diagnostic tests have now been developed, but these are only now being tested in the field (Slater et al. 2015). Such tests have the potential to be much quicker but they are not as sensitive as the laboratory based tests, and it possible that up to half of asymptomatic carriers will be ‘missed’. However, the contribution to malaria transmission of individuals with very low parasitaemias at the time of testing is uncertain, and the results of studies of the field effectiveness of MSAT with highly sensitive RDTs are awaited with interest. If MSAT with highly sensitive RDTs does turn out to be effective, effective community engagement will be as important as it is with MDA.

### Vector Control

The distribution of insecticide treated nets (ITNs) in Africa has had a major impact on malaria there, and as a result it has become almost an article of faith in the global malaria community that ITNs should be considered the most important single intervention in the battle against malaria (Bhatt et al. 2015). In a WHO-sponsored meeting on tackling artemisinin resistant malaria in the GMS the chairman suggested that *all* the additional resources being made available to counter resistance in the region should be spent on ITNs (*NPJD personal communication*). Unfortunately, Southeast Asian malaria vectors and populations do not behave like African vectors and populations, with most transmission occurring in the forest rather than in dwellings (Gryseels et al. 2015; Smithuis et al. 2013a). Several studies have now confirmed the limited efficacy of bed nets in malaria elimination effort in this region (Smithuis et al. 2013b; Satitvipawee et al. 2012). An important ethical issue here is around resource allocation, and overcoming established (but not evidence-based) pro-ITN sentiment amongst international and national policy makers. ITNs do have an important role to play and are relatively cheap to deploy, but given the evidence of differences in vector behaviours in Africa and the GMS, the relative allocation of limited resources should be driven by evidence-based health economics studies (Drake et al. 2015).

### Targeting ‘Source’ Populations

In the GMS, malaria transmission is concentrated in poor, hard-to-reach, highly mobile populations. Transmission is mainly occupationally related, highest among men who travel into forested areas to work. Many of the most at-risk populations are disenfranchised minority groups, often living in border regions, with little or no health infrastructure. Understanding the drivers of transmission in these populations entails acquiring better knowledge of population movement/migration, much of which is ‘illegal’. Working with these populations requires sensitivity not only to the cultural contexts but also to the uncertain legal status of many of the individuals. Several of the more endemic areas are mired in armed conflicts, and many populations are vulnerable as refugees or economic migrants without papers. The area currently with the highest endemicity in the GMS, for example, is Rakhine State in Myanmar, currently undergoing considerable civil strife and large-scale movement of populations. NGOs, government workers and researchers have to work within their own externally determined constraints, limiting their ability to engage ‘source’ populations. Even if the not inconsiderable task of eliminating malaria from many of these areas were to be achieved, political difficulties, the mobile nature of these populations, and changes in the ability to access them would leave them vulnerable to the reintroduction of malaria. Furthermore, as immunity will have waned because of the intervention the public health consequences of this reintroduction could potentially be worse than if malaria had not been eliminated in the first place.

### Mandatory Screening

There are a number of situations in which mandatory screening for asymptomatic malaria may be indicated to prevent individuals unwittingly spreading drug resistant malaria parasites. Following the disastrous importation of cholera into Haiti by African UN peacekeepers (leading to 8300 deaths), for example, a call has been made for Southeast Asian Peacekeepers to be screened for malaria before they travel to missions in Africa (Houston and Houston 2015). This would prevent peacekeepers from importing drug resistant malaria to a drug sensitive region, and could be implemented by the UN. Another situation where mandatory screening could potentially be introduced in the context of eliminating artemisinin-resistant malaria within the GMS, would be screening local people moving between areas where malaria has and has not been eliminated. Although practically difficult to implement, this has the potential to be of real importance in geographical locations with highly mobile populations – such as along the Thai-Myanmar Border. There are a number of practical barriers to implementing such a policy, which make it unlikely to be introduced at present. However, its possibility raises important ethical questions about the legitimacy of overriding personal autonomy in the global public interest and its limits.

### Triple Artemisinin Combination Therapies (TACTs)

The rationale for TACTs is similar to that of triple or quadruple therapy in HIV, tuberculosis, leprosy and other infectious diseases – to prevent the development of resistance. Two TACTs are currently being studied in 16 sites in Asia and 1 site in Africa (web identifier: NCT02453308): dihydroartemisinin-piperaquine + mefloquine; and artemether-lumefantrine + amodiaquine. The combination of a short acting artemisinin with two long acting partner drugs ensures that parasites are less likely to encounter only one long acting partner drug at any one time, minimizing the chance of resistance developing. In addition, it is hypothesized that these triple therapies could exploit potential inverse relationships between the parasite molecular resistance mechanisms to the paired long-acting partner drugs. It is thought that the wide implementation of triple therapy in malaria will slow the spread of multidrug-resistant malaria in areas where artemisinin and partner drug resistance is well established, and slow down or prevent the emergence of drug resistance in areas where resistance has not yet emerged. It is in the latter case where TACTs should be most effective.

There are several ethical issues to be considered here. TACTs differ from other examples of combination therapy in that the objective is to prevent antimalarial drug resistance at the population rather than at the individual level. Unlike in chronic infections such as TB and HIV development of ACT resistance within an individual patient during treatment is rare. Hence individuals are potentially exposed to the additional side effects of three rather than two drugs for little or no benefit to themselves; it is against the interest of the individual patient (usually a child) to take three rather than two drugs. If the strategy works the benefit will be to the population, which will only indirectly benefit the individual. In addition, TACTs are expected to be most effective at countering resistance in areas where resistance has not yet developed to any of the components, so that the long acting partner drugs will protect each other from the development of resistance and both will protect the short acting artemisinin component. Hence the areas where they will be most effective will be the ones where currently ACTs remain highly effective at the individual level.

Preliminary evidence of triple therapy is promising but safety and efficacy data are not widely available yet. Even if evidence is available, populations where ACTs still work such as in Africa where the majority of malaria cases are in children under five, may not readily change their prescribing behaviours. Other practical problems might be access to the triple therapy, availability of co-formulated drugs and the problem of substandard and falsified drugs especially in the private and informal sectors (Liverani et al., Chap. 10.1007/978-3-030-27874-8_5, this volume).

## Summary of Ethical Considerations

Above we have attempted to illustrate the ethical complexity of the implementation of the strategies widely agreed by experts to be necessary for the control of resistance to antimalarial drugs. It is clear that there are strong ethical arguments in favour of the implementation of such strategies in the global public interest. However, the considerations outlined above suggest that such interventions raise important ethical questions both about the nature and scope of implementation itself and about the obligations of countries both outside and within the region to those who are to bear its costs. The success of an elimination strategy based on these elements will depend upon these problems being addressed. In the remainder of this chapter, we summarise what we consider to be some of the most important ethical tensions and outline some preliminary thoughts about ways these might be addressed.

### Autonomy and Consent Versus the Global Benefit

There are a number of different ways in which the implementation of the intervention strategies above raises important questions about respect for autonomy. In some cases, these interventions may lead to tensions between the interests and wishes of individuals and the global benefit. During implementation of each strategy, individuals in selected communities are subjected to interventions – ‘treatment’ or surveillance – not for their own good but for the common good of current and future populations both locally and internationally (see discussion on common goods in Chaps. 10.1007/978-3-030-27874-8_8 and 10.1007/978-3-030-27874-8_9) (Jamrozik and Selgelid, Chap. 10.1007/978-3-030-27874-8_1, this volume-a; Smith and Coast, Chap. 10.1007/978-3-030-27874-8_17, this volume). Under current circumstances, such interventions are voluntary: making MDA compulsory or imposing travel restrictions on people who have come from areas with artemisinin resistant malaria is not considered achievable or justified at present. However, it is possible that as in other global health contexts this judgement might change and that individuals may lose their right to opt out of, for example, MDA. Draconian measures have been taken to contain dangerous contagions such as H5N1 influenza, SARS, MERS-CoV, Ebola virus, Lassa fever, and multidrug-resistant tuberculosis, which involved restriction of liberty in order to protect the public. Were compulsory approaches to be considered in the context of malaria, this would present important questions about the legitimacy of restrictions of liberty per se but also questions about how this was in fact undertaken, and about the nature of the obligations of the wider global community – particularly wealthy countries – to those who are subjected to such interventions in the global health interest.

Questions about autonomy also arise in the context of voluntary approaches. Where individuals and their communities are being asked to decide about participation in the strategies outlined above, it is vital that best efforts are made to ensure that any consent they give is grounded in a good understanding of the implications. However, the evidence is that valid consent is likely to be difficult to achieve in such contexts. This places particular importance on the roles of wider communication, community participation, political involvement and other forms of public engagement preceding and during the intervention. It has to be acknowledged that even in the context of well-resourced, evidence-based approaches to consent and community engagement, understanding is likely to be partial given the complexities of malaria transmission and how these inventions work. This need not mean that the choice to participate is invalid but it does mean that the moral basis for the intervention cannot rest on consent alone, even when the choice to participate is voluntary. This suggests that those who are responsible for the conduct of such strategies have obligations to ensure that they are conducted to high ethical standards, and that appropriate protections, and possibly compensation, are in place.

### Risk Benefit

The potential benefits of malaria elimination are substantial, including the direct burden averted and economic growth through improved educational attainment and productivity; these gains were estimated recently to far outstrip the costs required to achieve them (Purdy et al. 2013). That said it is clear from the discussion above that those who bear the consequences of malaria elimination efforts are not those who will benefit directly. The majority of the populations with the highest prevalence of resistant malaria and of submicroscopic malaria in Southeast Asia are poor and mobile forest workers (Phommasone et al. 2016; Tripura et al. 2017). This is unsurprising as vulnerability to malaria – as is also true of many other infectious diseases – is largely a consequence of social determinants of health such as poverty, malnutrition and insufficient access to healthcare. Malaria burden is both a consequence and an illustration of global inequities. These populations are already burdened by their circumstances and environments. Yet they are the very individuals who will likely to be shouldering the burdens of any global intervention to curb resistant malaria. In the case of MDA, entire communities are treated whether or not they are unwell with malaria. That means that many individuals who are neither ill nor carriers of the parasite will be asked (or required) to take drugs and therefore be at some risk of potential adverse drug reactions. This uneven distribution of individual risks and inconveniences – that is individuals in SEA shouldering the burden for the benefit of good health outcomes primarily in the interests of populations elsewhere – is a key moral challenge. Whilst at the macroeconomic level the costs of malaria elimination are outweighed by the benefits, this may well not be true at the level of the individuals involved. Important ethical questions concern the question of when, if at all, the imposition of risks of harm on (often vulnerable) individuals is legitimate in the interests of others and the limits of this. The ethical questions here concern not only those related to whether the imposition of such burdens is justified but also, where this is the case, both the approach adopted to such implementation and the nature and scope of our obligations to those upon whom it is imposed. Is there, for example, an obligation to compensate such populations?

### Data and Sample Sharing

Ethical issues also arise with regard to the international collaboration required to ensure high scientific and public health standards in the interventions. This is important because it is the achievement of such high standards (and hence the potential for success) that justify the imposition of risks and restrictions of liberty on vulnerable populations. In order to make the most informed decisions about planning interventions to eliminate malaria, there is a need to ensure that there is access to as much good quality data as possible. That is, it depends crucially upon data sharing. However, there is generally a lack of transparency and confidence in the quality of available malaria data. This can be due to poor quality data and underreporting of cases, which can in turn be due to variable availability of diagnostic tools such as rapid diagnostic tests and blood slides, unsurprising given that data are frequently collected under resource-starved conditions. This contributes to the lack of trust in the data on antimalarial resistance, and in the data used in mathematical modeling and the resulting predictions. An additional key problem is that many national malaria control programmes do not readily share their malaria data for political, economic and national security reasons. Data related to population movement and migration that could aid interventions such as MDA and engagement with “source populations” are, for example, particularly difficult to access. Although there is advantage in data sharing, it is also acknowledged that it can pose a number of ethical challenges around issues of privacy, potential stigma and economic harms (Mishra et al. 2016; Bull et al. 2015; Cheah et al. 2015). This suggests that questions about the ethical tensions between the interests of individuals and the global health interest also arise at the level of institutions, health ministries, and countries.

### Scientific Disagreement About the Best Way Forward

We outlined a number of proposed interventions above, and given the current state of the evidence there are valid debates in the scientific community about what action or actions are appropriate where. Interventions such as TACTs and MDA are still under study. An important, as yet unresolved, scientific debate is about the way resistance spreads or emerges. There are data supporting both sides of the argument – geographical spread of sporadic vs. spontaneous distributed emergence (Lu et al. 2017). However, there is also a limited window of opportunity to act. There are strong moral reasons for acting to prevent the spread of resistance both within and beyond Southeast Asia. Inaction will almost inevitably lead to a repeat of history – the loss of safe, inexpensive and highly effective treatments and an increase in cases of severe malaria and related deaths. It may also mean that a once in a generation opportunity, capitalizing on the combination of the availability of political will and effective tools to take a big step towards the eradication of malaria, will have been missed. There remains, however, a degree of scientific uncertainty. This raises ethical questions about the level of scientific consensus required for action. Understanding is likely to remain imperfect. Is it legitimate to initiate strategies such as those outlined above on the basis of good but imperfect understanding? The answer to this question cannot be ‘never’ because there is widespread agreement about the urgency of the situation and a residual degree of scientific uncertainty will always remain.

## Conclusions

In this chapter, we have described practical ethical issues arising in currently proposed interventions (and the lack of them) to reduce the risk of the movement of resistance to the current best antimalarial drugs from Southeast Asia to Africa, as well as to prevent resistance emerging in Africa. Whilst strong ethical reasons for such interventions are provided by the seriousness and scale of the threat and the existence of a degree of scientific consensus on this strategy, its implementation is ethically complex. We have outlined some of the most important practical ethical problems presented by each of possible components of the proposed strategy, and have also argued that even if the various interventions were to be ethically justified this would not be the end of the ethical debate. We have argued that important ethical questions about the mode of implementation of the interventions and about the obligations of the wider community to those they affect would remain.

Some of the most important of such considerations are those relating to fairness in the selection of interventional populations. All populations which meet a set of criteria for an intervention, be it MDA, TACT or travel restrictions, should where practical have the same intervention. The intervention should be evidence-based and justified, and all relevant stakeholders should be involved in the decision-making process and have meaningful input into the deliberations. The manner and context in which decisions are made should be reasonable, fair and transparent.

In addition to ethical considerations relating to the selection of such populations, we have argued that important obligations exist for those countries and governments that can afford it to assist and perhaps to compensate those individuals who are subject to such interventions and may experience harms as a result of their participation (Upshur 2002). Meeting these obligations may call for the provision of compensation, for example where businesses suffer due to lack of mobility or where people suffer from side effects of MDA. Or perhaps, in the form of community level benefits such as improved healthcare facilities. An important aspect of the obligations of the wider world to those who live in the region is that any intervention is well-planned and adequately resourced. It is clear that curbing antimalarial resistance, similar to resistance of other antimicrobials, is both a global priority and a global responsibility (Jamrozik and Selgelid, Chap. 10.1007/978-3-030-27874-8_1, this volume-b). Both scientifically and in terms of effective public health interventions, solutions to this problem are inevitably going to be collaborative. In addition to the provision of adequate resources, communities, countries, researchers and funders must be encouraged to work together. It is our view that four key requirements for a successful and appropriate collaborative approach to addressing emerging ACT resistance, and hence ethically important requirements of those who propose such interventions, are as follows:

i. *Encouraging and funding more research*. Research should be conducted to address the gaps needed for each of the interventions proposed by the scientific community such as determining the safety of DP for MDA, the efficacy and safety of triple therapy, and determining the way resistance spreads. More evidence would help channel resources to the correct people and places, and facilitate a scientific consensus.
ii. *Retraining and supporting village malaria workers* so that they are able to manage other febrile diseases and hence remain relevant and retain community support. This could be provision of education and strengthening support from provincial health departments.
iii. *Encourage collection of quality malaria data, and sharing and pooling of these data*. A data sharing initiative, the WorldWide Antimalarial Resistance Network (www.WWARN.org) was established by malaria researchers in 2009 to facilitate collaborative study groups working to answer specific research questions using pooled analyses. WWARN has had considerable success in pooling individual-participant data from multiple clinical trials from academic groups and pharmaceutical companies, but has been less successful with NMCPs. Individual research groups have also established data sharing mechanisms via a managed access route (Cheah and Day 2017).
iv. *Engaging with affected communities in creative and sensitive ways*. Some work has already been conducted to engage forest workers, minority groups and mobile populations, and much more is needed (Lim et al. 2017). This will improve understanding of the science behind malaria and malaria elimination and will facilitate interventions such as MDAs and MSATs.
